# Insights into the Genital Microbiota of Women Who Experienced Fetal Death in Utero

**DOI:** 10.3390/microorganisms11081877

**Published:** 2023-07-25

**Authors:** Mira Holliday, Kumar Uddipto, Gerardo Castillo, Luz Estela Vera, Julie A. Quinlivan, George L. Mendz

**Affiliations:** 1 College of Health and Medicine, Australian National University, Canberra, ACT 2601, Australia; mira.holliday@anu.edu.au (M.H.); julie.quinlivan@nd.edu.au (J.A.Q.); 2 School of Medicine, Sydney, University of Notre Dame Australia, Darlinghurst, NSW 2010, Australia; kumar.uddipto@nd.edu.au; 3 Área de Ciencias Biomédicas y Policlínico, University of Piura, San Eduardo, Piura 20009, Peru; gerardo.castillo@udep.edu.pe (G.C.); luz.vera@udep.edu.pe (L.E.V.); 4 Institute for Health Research, University of Notre Dame Australia, Fremantle, WA 6160, Australia

**Keywords:** bacterial taxa, culture-independent techniques, amplicon sequencing, vaginal microbiota, amniotic microbiota, preterm birth (PTB), stillbirth

## Abstract

The aim of this work was to achieve a better understanding of the bacterial pathogens associated with stillbirths that would serve to inform clinical interventions directed at reducing this adverse pregnancy outcome. A prospective observational study was conducted with the participation of 22 women from northern Peru, of whom 11 experienced fetal death in utero and 11 delivered preterm births. Swabs were taken from the vagina, placenta, amniotic fluid and axilla of the infant at birth by Caesarean section. The bacterial populations in the vagina and the amniotic space of each participant were determined by employing the amplicon sequencing of the V4 region of the *16S rRNA* genes. The sequence data were analysed using bioinformatics tools. The work showed differences in the composition of the genital microbiomes of women who experienced preterm birth or fetal death in utero. There were no differences in the alpha diversity between the genital microbiotas of both groups of women, but there were more different taxa in the vagina and amniotic space of the preterm participants. *Lactobacillus* spp. was less abundant in the stillbirth cases. *E*. *coli*/*Shigella*, *Staphylococcus*, *Gardnerella*, *Listeria* and *Bacteroides* taxa were associated with the stillbirths. In each woman, there was a minimal concordance between the bacterial populations in the vagina and amniotic space.

## 1. Introduction

Fetal death in utero (FDIU), also referred to as a stillbirth, is a serious adverse pregnancy outcome. It is generally defined across the developed world as the death of the fetus after 20 completed weeks of gestation [1], but in developing countries can be defined as fetal loss after 22 or 28 completed weeks of pregnancy with a fetal weight of at least 500 g or 1000 g, respectively [2]. The inconsistent definition contributes to the difficulty in capturing complete data, impacting the understanding of its aetiology [2].

In 2019, an estimated 1.9 million infants were stillborn, with a global rate of 13.9 stillbirths per 1000 total births [3], with large regional variations from 22.8 stillbirths per 1000 total births in West and central Africa to 2.9 per 1000 in western Europe [4]. Global rates decreased from 21.4/1000 births in 2000, owing to improved access to antenatal care and increasing knowledge surrounding maternal risk factors [4]. In 2020, the Australian stillbirth rate using the definition of loss after 20 weeks gestation was 6 per 1000 births [5].

Common causes of stillbirths include infections, birth defects and pregnancy complications, such as pre-eclampsia or diabetes [6]. More than 10% of stillbirths are due to infection in the fetus, placenta or mother. Infection as a causal aetiology increases in stillbirths after 24 weeks gestation. The rate of pregnancy loss varies with gestational age at the onset of infection and by the specific pathogen. For example, infection with *Treponema pallidum* causes pregnancy loss or fetal death in up to 50% of cases, whereas parvovirus B19 infection causes pregnancy loss or stillbirth in less than 3% of cases [7]. The mechanisms of pregnancy loss owing to pathogens known to traverse the maternal–fetal barrier and cause congenital disease in the fetus can be directly pathogen-mediated, placenta-mediated and/or through inflammation-induced previable delivery [7]. It is important to determine the cause(s) of death to prevent adverse events in future pregnancies. Bacteria linked to stillbirth include *Streptococcus agalactiae*, *Escherichia coli*, *Haemophilus influenza*, and species of the genera *Klebsiella*, *Enterococcus*, *Chlamydia*, *Mycoplasma* and *Ureaplasma* [8].

The vaginal and intra-amniotic microbiomes play an important role in the health of the maternal reproductive tract and have roles in the physiology, immunity and reproductive success. *Lactobacillus* species are present in the vaginal microbiota of almost every woman. The vaginal microbiome is a dynamic microenvironment, where the relative abundances and frequencies of species depend on various factors, including pregnancy, contraceptive use, sexual activity and menstrual cycles, as well as the racial background of the woman [9,10]. The genus *Lactobacillus* is found as the predominant commensal bacteria in the healthy human vagina [11]. The dysbiosis of this microbiome is most notably associated with the development of infections due to bacteria [12] and other pathogens [13].

The vagina can be the source and pathway for the ascending colonisation of the uterus by pathogens; hence, the presumed associations of vaginal dysbiosis with miscarriage [14,15,16], low birth weight [12] and preterm birth [17,18]. A limited number of studies have evaluated the relationship between bacterial dysbiosis and the occurrence of stillbirth, but infection with pathogens has been linked with fetal death, including infection with *S*. *agalactiae* [19], *Chlamydia*, *Enterococcus*, *E*. *coli*, *Klebsiella*, *Mycoplasma*, *Listeria monocytogenes* and *Ureaplasma urealyticum* [7,8]. There are conflicting reports about the existence of a microbiome in the intra-amniotic space; however, it is a known site for potential pathogen colonisation that has been causally linked to adverse outcomes in pregnancy [20]. Additionally, microbiomes of the meconium [21,22] and amniotic fluid have been identified [23,24] and implicated in the diagnosis of chorioamnionitis [25] and the premature rupture of membranes [26], both adverse conditions are linked to an increased risk of stillbirth.

This prospective observational study compared the genital microbiota of women who gave birth preterm (PTB) versus those with the adverse outcome of stillbirth, with the aim of identifying taxa that may be associated with stillbirth. The DNA profiles of bacterial communities in the vagina and the amniotic space were investigated by employing the 16S rRNA gene sequencing of DNA extracted from swabs of the vagina, placenta, amniotic fluid and axilla of the infant at the moment of birth by Caesarean section. Protocols to minimise reagent-based and environmental contaminations were applied. The information on potential associations of taxa with stillbirth could serve to devise strategies to detect, manage and reduce this adverse pregnancy outcome.

## 2. Methods

### 2.1. Participants

Participants were recruited as part of a large prospective observational investigation from the Cayetano Heredia Hospital, Piura, northern Peru. Women in this study represented a subgroup of those who gave consent to participate in a larger investigation about PTB. All women in both groups had emergency procedures with decision to delivery intervals of less than 24 h. A reason as to why these women had Caesarean deliveries was that they had a previous Caesarean section, and the hospital where they were recruited did not perform a trial of labour after a uterine scar; in this setting, the obstetric team elected for Caesarean sections.

The participants answered a questionnaire requesting information on their age, racial background, gestational age, signs of genital infection during the pregnancy and indicators of infant health. Inclusion criteria were women experiencing FDIU, with or without symptoms or signs of infection at delivery and with unknown causative microorganisms.

The control group included participants of the larger study who did not experience a stillbirth but delivered a preterm infant without secondary complications. The selection of preterm controls was partially to adjust for the impact of gestational age on the microbiome, which is known to stabilise midgestation. An inclusion criterion was delivery by Caesarean section in order to reduce the risk of the potential contamination of the microbiome, owing to labour and vaginal delivery. Thus, FDIU and PTB cases where vaginal delivery was able to be performed were excluded (Table S1).

### 2.2. Ethics Approval

The participants were recruited on a voluntary basis and provided written informed consent. Women in this study recruited in Peru had all their information materials written in their local language. The main study was approved by the NSW Health Western Sydney Local Health District Human Research Ethics Committee (HREC#2012/9/4.6(3490)) as the coordinating site. The local and international sites had a cross-institutional ethics approval.

### 2.3. Sample Collection

The patients were cared for following the Social Security protocol for Public Hospitals in Peru for women at risk of preterm delivery. Samples were always obtained before the administration of antibiotics. Vaginal swabs were collected from the vaginal midpoint (3 cm from introitus) using a sterile plastic swab with a Dacron tip immediately prior to delivery. All women required Caesarean sections and the same sterile plastic swabs were employed to collect intrauterine samples. Amniotic fluid swabs were collected under surgical field sterile conditions at the time of uterine incision at Caesarean section. Axilla swabs were collected from infants using a sterile technique immediately after delivery from the uterus. Placental swabs were collected under sterile conditions by swabbing between the amnion and chorion layers of the placenta. Two swabs were serially collected from each site for quality assurance purposes. All samples were obtained under sterile conditions by a qualified health professional, labelled for their participant origin, deidentified from the participant and immediately preserved at −18 °C in sterile tubes until DNA extraction.

### 2.4. DNA Extraction, Amplification and Sequencing

From the individual swabs collected in duplicate, genomic DNA was isolated and purified employing the QIAamp DNA Mini Kit (QIAGEN, Chadstone Centre, Clayton, VIC, Australia), as per manufacturer protocol; the concentration and quality of the DNA were assessed using a Nanodrop ND-1000 Spectrophotometer (Nanodrop Technologies; Wilmington, DE, USA). The composition of the microbial communities was determined at the Alkek Center for Metagenomics and Microbiome Research (Houston, TX, USA) with high-throughput sequencing of the PCR amplified V4 region of the 16S rRNA gene using the barcoded Illumina adapter-containing primers 515F and 806R with 2 × 250 bp cartridges in a MiSeq instrument (Illumina, San Diego, CA, USA) [27]. Negative controls were included for each DNA extraction kit. The sequences were clustered into operational taxonomic units (OTU) at a similarity cut-off value of 97% using the UPARSE algorithm [28]. Chimeras were removed using USEARCH v7.0.1090 and UCHIME. To determine the taxonomies, the sequence reads were quality checked, trimmed using the features of QIIME [27] and aligned against the SILVA reference database using standard operating procedures [29]. The samples were rarefied to 4000 reads and rarefaction resulted in the loss of all negative controls (Table S2).

### 2.5. Data Analyses

Analyses of the sequence data yielded the number of reads (abundance) and the identification of the bacterial taxa (diversity) for each sample of each participant. Taxon abundance was determined from sequence counts and expressed in percent. Analyses of the relative abundance of genera at different locations were limited to those with average abundances greater than 1%. This served to minimise the problem of including the genera of bacteria from potential contaminants.

A principal component analysis (PCA) was carried out on the dataset where the taxa relative abundances measured in each sample of each participant had been dimensionally reduced and trimmed. All taxon genus data with zero variance across samples were trimmed and removed in order to prepare the data for the PCA. The advantage of the PCA is that it completely removes random correlations resulting in datasets and reduces the statistical overfitting of data. The main disadvantages of this type of PCA calculation are that independent variables become less interpretable and the requirement for data to be standardised before conducting the analysis, which often means that some loss of data is liable to occur.

Genital taxa sequence data were analysed to determine species diversity and richness, and Shannon’s diversity index was used for each participant and the entire cohort. The diversity index was calculated using the Shannon–Wiener expression:

H = −∑ p_i_ * ln(p_i_)

where p_i_ is the proportion of each taxon in the sample.

Richness (T) was the total number of different taxa in a sample. Evenness was calculated as:

J = H/ln(T)

The analysis of bacterial communities was conducted in R [30]. To calculate the alpha diversity and taxon relative abundance, the Kruskal–Wallis test [31] was applied to determine the overall statistical significance of the two groups. If the Kruskal–Wallis test yielded a *p* < 0.05, pairwise significance was found based on the Mann–Whitney test [32]. Differences in taxa beta diversity were ascertained using PERMANOVA. Linear regressions were performed using the lm function in R. To compare more than one measure, such as multiple measures of alpha diversity or multiple taxonomic genera, *p*-values were adjusted for multiple comparisons with the false discovery rate algorithm [33]. After removing confounders, the linear discriminant analysis effect size (LEfSe) method [34] was applied to compare taxa enrichment between FDIU and PTB women in the vagina and amniotic space.

The concordance between the presence of taxa in the vagina and the amniotic space was evaluated by analysing the sequence data for each woman in both locations. The concordance was absent for taxa identified only in the vagina or the intra-amniotic space, partial concordance was that of taxa present in the vagina and 1 or 2 of the intra-amniotic space swabs and full concordance was of vaginal taxa found in all intra-amniotic swabs.

## 3. Results

### 3.1. Characteristics of Participants

A total of 22 mixed-race patients from Peru were recruited in the study, with 11 experiencing FDIU and 11 controls delivering preterm. Participants in each group were closely matched for age, gestational age and gravidityMetadata are summarised in Table 1.

There was no significant difference in the mean age of women in the preterm and FDIU groups (*p* > 0.05); the mean gestational age at delivery was different in both groups (*p* < 0.05). No woman in the study was nulliparous. Five women in the PTB group and seven women in the FDIU group experienced genital infections during pregnancy, which included symptoms such as fever, vaginal discharge, bleeding and pelvic tenderness, with no causative microorganism identified in either group. There were no differences in the number of complications associated with stillbirth such as pre-eclampsia and the preterm premature rupture of membranes; other complications were present only in single instances and there were similar numbers in both groups. The FDIU cases did not have a cause identified for the stillbirth (Table 1).

### 3.2. Composition of the Microbiomes

**Figure 1:**
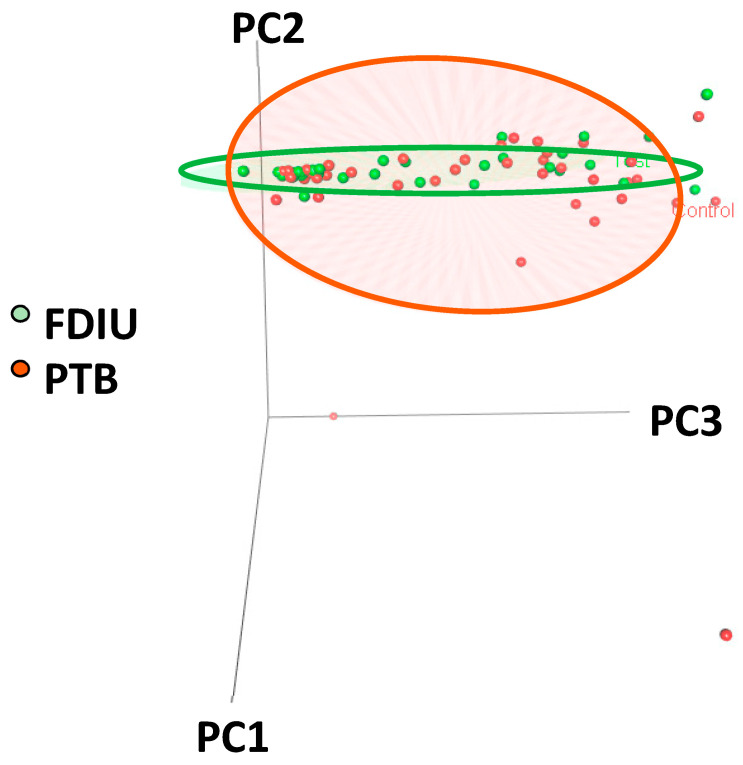
Principal component analysis of the taxa relative abundances comparing the composition of microbiomes of women who delivered stillbirth (green dots) and PTB (brown dots). The ellipsoid contours enclose 50% of the data of each group.

The composition of the microbiomes of women in the FDIU and PTB groups was investigated by employing the principal component analysis (PCA) of the relative abundances of taxa in each woman. The results of the analysis indicated greater variance in the control populations than in the FDIU populations; a 3D plot showed that, overall, they were different (Figure 1). The results of the PCAs of the vaginal and amniotic microbiomes of both groups of women showed that they were similar for vaginal microbiota (Figure S1) and different for amniotic microbiota, with greater variances in the control populations (Figure S2).

### 3.3. Composition and Diversity of the PTB and FDIU Genital Microbiomes

Analyses of the diversity of the genital microbiome samples of both groups of women examined the number of OTU and taxa (richness), the distribution of relative abundances of taxa (evenness) and the number of taxa and the inequality between their abundances (Shannon diversity index).

Table 2 summarises the diversity values of the genital samples comparing PTB controls and FDIU cases; in each group, the number of taxa in the vagina was significantly smaller than in the amniotic space. Between groups, the difference in the richness of the vaginal taxa was small, but richness was 20% greater in the PTB amniotic taxa. In the PTB and FDIU groups, there was no significant difference between the Shannon index for the vaginal or amniotic microbiomes, *p* = 0.190 and *p* = 0.944, respectively, albeit the number of taxa in the vagina was significantly smaller than in the amniotic space (Table 2).

### 3.4. Relative Abundance and Frequency of Taxa

In both groups of women at the taxon level, the LEfSe analyses showed several taxa with enriched relative abundance in the vagina and the amniotic space. The analyses yielded the genera *Bifidobacterium*, *Geobacillus* and *Thermomonas* enriched in the vaginas of women who experienced FDIU; this result stemmed mainly from these taxa found at relatively low abundances in women of the control group, with *Bifidobacterium* and *Geobacillus* being absent from the vagina of all the PTB women and *Thermomonas* being present in only one control woman. In the amniotic space, the genera *Prevotella* and *Dialister* were more abundant in women who delivered FDIU, but at low relative abundances. To verify these results, a multiple comparison correction was performed and no q-values were significant.

**Figure 2:**
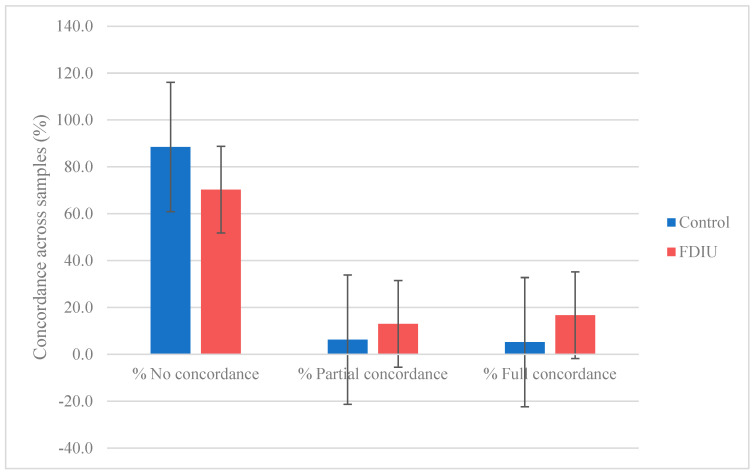
Concordance of the presence of taxa between vaginal and amniotic samples.

Comparing the taxa present in the paired vaginal–amniotic space samples of each woman in the study, there was no concordance between the most taxa in the vagina and the amniotic space in PTB and FDIU samples (Figure 2). Partial or full concordance instances were more common in FDIU samples (Figure 2). These differences in the data supported the ruling out of the significant contamination of the amniotic samples.

To better understand the contribution of individual taxa to fetal demise, data were extracted on the frequencies and relative abundances of the 12 most abundant taxa present in FDIU patients. Table 3 shows the taxon frequencies and abundances of these 12 taxa in the vagina and amniotic space for both groups of women. We noted the frequencies and abundances of *E*. *coli*/*Shigella*, *Staphylococcus*, *Gardnerella* and *Listeria* in the amniotic space of FDIU samples. On both measures, these potentially pathogenic taxa were found in greater abundances in FDIU than in PTB patients, and at equal or higher frequencies.

*Listeria* taxa were present at high abundances in the vaginas and amniotic spaces in the samples of two women (18%) in the FDIU group and were completely absent in the control samples. In the FDIU group, *Gardnerella* had medium abundances in the vaginas of four women (36%) and high abundance in the amniotic spaces of two women, whereas in the controls, it had high vaginal taxa abundance and low abundance each in one woman, and medium abundance in the amniotic spaces of two women. *Staphylococcus* was found in the amniotic spaces of the FDIU group at high abundance in two women and low abundance in eight women (73%); in the vaginas of women in the same group, it was found at high, moderate and low abundance in one, one and four women, respectively. *Staphylococcus* was present at high abundance and low abundance in the vaginas of each of two women in the control group, in medium abundance in one woman and low abundance in seven women (64%). In the FDIU group, high, medium and low abundances of *E*. *coli*/*Shigella* were detected in the amniotic spaces of one, five and two women, respectively, and low abundance in the vaginas of six women (54%). This taxon was absent in the vaginas of the women in the control group, and the amniotic spaces included mostly women with low relative abundances of *E*. *coli*/*Shigella*. The other eight taxa were present mostly in low abundances in the vaginas and amniotic spaces of both groups of women (Table 3).

The dominant taxon in the amniotic space of some individual FDIU patients supported their potential associations with stillbirth. *Listeria* and *Staphylococcus* were each dominant in two women and *Gardnerella* and *E*. *coli*/*Shigella* were dominant each in one woman. In addition to the taxa identified in the collective analyses of the microbiomes, *Bacteroides* was dominant in the vagina and amniotic space of one woman and present only at low abundance in the amniotic space of one PTB patient.

Other pathogenic taxa, such as *Mycoplasma* and *Ureaplasma*, that could cause infections in the genital tract were identified only in several FDIU and PTB patients and mostly at low concentrations. *Mycoplasma* was present at high concentrations in the amniotic space and vagina of one FDIU and one PTB patient, respectively. The frequencies and abundances of these taxa did not make it possible to establish an association with stillbirth.

### 3.5. Lactobacillus Relative Abundances

None of the 22 women in the study delivered at term, suggesting some degree of dysbiosis in their microbiota. Healthy genital bacterial populations are commonly dominated by *Lactobacillus* spp. These taxa were ~3 times more abundant in the vaginal microbiota of women in the PTB group; using a threshold of 50% relative abundance as a criterion for dominance, *Lactobacillus* taxa in the vagina were dominant in one FDIU patient and in five PTB women. *Lactobacillus* taxa in the microflora of most intra-amniotic samples had very low relative abundance and in only one PTB sample had a medium abundance.

## 4. Discussion

The clinical metadata did not provide firm insights into the potential infectious agents of the stillbirths (Table 1). To search for associations of taxa present in the genital tract with stillbirths, the vaginal and amniotic microbiomes of women who delivered FDIU or PTB were compared employing culture-independent high-throughput sequencing and bioinformatics analyses. The study investigated the presence of taxa specifically associated with stillbirths as opposed to those present in live births of preterm infants. The participating women were from northern Peru and had similar mestizo racial backgrounds, which set aside potential natural variations arising from racial background and some environmental factors [9,35].

The results of the principal component analyses suggested a difference in the overall composition of the vaginal and amniotic microbiomes between both groups of women, with taxa in the PTB cases being more scattered in the 3D PCA plot (Figure 1). This conclusion was supported by the results of the analyses of the diversity of the microbiomes (Table 2). Separate comparisons between the microbiota in the vagina and intra-amniotic space compartments also showed differences between the PTB and FDIU women for each location, an outcome similar to the previous finding that there were differences in microbiome compositions in miscarriage cases compared to healthy deliveries [14,15]. Since stillbirths were compared to PTBs, these results underline the role of the microbiome in the demise of the infant. No significant diversity was measured between the microbiotas of both groups suggesting a role for specific taxa in FDIU (Table 2) rather than the overall characteristics of the microbiomes.

Selecting the 12 taxa with the greater relative abundances in the amniotic microbiomes of women who experienced FDIU, their frequencies and abundances were determined in the vaginas and amniotic spaces of women in the PTB and FDIU groups (Table 3). In the latter group, most of these taxa were identified at low (1–9%) or medium (10–49%) relative abundances and/or low frequencies in the amniotic space, suggesting that individually by themselves they were unlikely to be associated with fetal death. However, the frequency and abundance of *E*. *coli*/*Shigella*, *Staphylococcus*, *Gardnerella* and *Listeria* in the microbiomes of women in the FDIU group, and the much lower values for the four taxa in the microbiomes of the women in the PTB group suggested a possible association with the outcome of fetal demise. Analyses of the microflora of individual participants supported this conclusion.

*E*. *coli*, *Staphylococcus* and *Gardnerella* can cause aerobic vaginitis and are considered to have a role in repeated pregnancy loss [36,37]. *E*. *coli* has been identified in the microbiome of women who delivered preterm [38]. Maternal *Staphylococcus* infections have been linked to PTB [39] and neonatal sepsis [40]. *Gardnerella* is present in the healthy vaginal flora of many women, but its overgrowth can cause bacterial vaginosis. It has been identified in the placenta [41] and associated with miscarriages [14] and PTBs [42]. *L*. *monocytogenes* is the causative agent of listeriosis, an infection that in the genital tract can be transmitted from mother to child through vertical transmission, transplacentally or during passage through the birth canal, lead to miscarriage, stillbirth, PTB, and congenital neonatal infections [43]. Fetal complications from *L*. *monocytogenes* often occurs in the absence of an overt illness in the mother, posing special risks for the infant. It is inhibited by the normally low vaginal pH, but could grow when it increases; thus, infants from women with raised vaginal pH values during pregnancy may be at a higher risk of listeriosis [44].

The dominance in the vaginal and amniotic space microbiomes of *Bacteroides* in a FDIU patient pointed to a potential involvement of this taxon in FDIU. *Bacteroides* is a common commensal of the gut, but is also a cause of serious invasive human infections [45], including genital infections [46], and of interactions with other genital pathogens [47].

The lower abundance and dominance of *Lactobacillus* spp. in the microbiomes of the FDIU women likely reflected a more pronounced dysbiosis in this group. This absence could have resulted in an increase in genital pH, allowing for the proliferation of pathogens such as *Listeria* that are inhibited at a normal low pH.

Other pathogenic taxa, such as *Mycoplasma* and *Ureaplasma*, that can cause infections in the genital tract were identified only in several FDIU and PTB patients, and mostly at low abundances. *Mycoplasma* was present at high concentrations in the amniotic space and vagina of one FDIU and one PTB patient, respectively. The frequencies and abundances of these taxa did not make it possible to establish an association with stillbirths.

In agreement with the results of this study, *E*. *coli*, *Staphylococcus* and *Listeria* were identified as pathogens present in the amniotic space in an investigation of the role of infections in stillbirths [48]. Additionally, a prospective study comparing the umbilical cord microbiomes of stillbirths and livebirths found six taxa uniquely present in stillbirths; of these, *Bacteroides* was the only taxon common within the findings of this work [49].

Evidence for the association with stillbirths of specific taxa present in the microbiota of women who experienced FDIU was found in this work. Larger studies would serve to verify these associations, perhaps even establishing causal links. This study also highlighted the importance of extending current microbiological hospital practices to screen for infectious agents by analysing vaginal swabs with culture-independent techniques that would serve to identify the presence of pathogenic microbes currently not associated with stillbirths. In addition, it is recommended that the histopathology of placentas is carried out to investigate microbiome histopathology correlations. These data would form the basis of interventions that could potentially prevent FDIU and other adverse pregnancy outcomes.

## Figures and Tables

**Table 1:** Demographic data and characteristics of participants’ pregnancies.

	PTB Group	FDIU Group
Number of participants	11	11
Mean maternal age (years and range)	29.1 (18–38)	30.2 (15–42)
Mean gestational age at delivery (weeks and range)	33.5 (30–36)	24.6 (23–31)
Parity		
1	5 (46%)	6 (55%)
2	4 (36%)	2 (18%)
≥3	2 (18%)	3 (27%)
Infection during pregnancy *	5 (46%)	7 (64%)
Complications		
None	5 (46%)	4 (36%)
Pre-eclampsia	1 (1%)	1 (1%)
Preterm premature rupture of membranes	1 (1%)	1 (1%)
Other **	4 (36%)	5 (46%)

* Includes symptoms such as discharge, fever, etc.; ** Includes single instances of gestational diabetes, hypertension, risk of abortion, etc.

**Table 2:** Diversity of the vaginal microbiome in PTB controls and FDIU cases. Number of OTU (S), number of taxa (T), richness (d), evenness (J) and Shannon diversity index (H).

Index	PTB Controls		FDIU Cases	
	Vagina	Amniotic	Vagina	Amniotic
S	210	379	194	353
T	38	147	44	111
d	3.637	4.990	3.784	4.709
J	0.322	0.599	0.613	0.643
H	1.172	2.991	2.319	3.028

**Table 3:** Overall frequencies of the 12 most abundant taxa present in FDIU patients. Data on the vagina and amniotic space are for both groups of women. Frequencies are shown as number of taxa/average abundance, the latter given as (+) 1–9%, (++) 10–49%, (+++) 50–100%. Absent (---).

Taxon	PTB		FDIU	
	Vagina	Amniotic	Vagina	Amniotic
*E*. *coli*/*Shigella*	---	1/(++); 7/(+)	6/(+)	1/(+++); 5/(++); 2/(+)
*Staphylococcus*	2/(+++), 2/(+)	1/(++): 7/(+)	1/(+++), 1/(++); 4/(+)	2/(+++); 8/(+)
*Gardnerella*	1/(+++); 1/(+)	2/(+)	4/(++)	2/(+++)
*Cloacibacterium*	2/(+)	3/(++); 5/(+)	1/(+)	8/(+)
*Enterococcus*	1/(+)	3/(+)	2/(++)	1/(+++); 3(+)
*Anoxybacillus*	1/(+)	10/(+)	2/(+)	10/(+)
*Streptococcus*	3/(+)	10/(+)	3/(+)	9/(+)
*Tepidomonas*	2/(+)	10/(+)	1/(+)	9/(+)
*Paracoccus*	1/(+)	6/(++); 3/(+)	1/(+)	8/(+)
*Listeria*	---	---	2/(+++)	2/(+++)
*Acinetobacter*	2/(+)	9/(+)	3/(+)	7/(+)
*Kocuria*	1/(+)	9/(+)	1/(+)	1/(++); 6(+)

## Data Availability

The data presented in this study are available in Tables S1 and S2 of the Supplementary Materials.

## Supplementary Materials

The following supporting information can be downloaded at: https://www.mdpi.com/article/10.3390/microorganisms11081877/s1, Table S1: Participant Metadata, Table S2: OTU Abundances. Figure S1: Principal Component Analysis of the Taxa Relative Abundances of the Vaginal Microbiomes Figure S2: Principal Component Analysis of the Taxa Relative Abundances of the Amniotic Microbiomes title.
