# Supplementary material for: Insights into the Genital Microbiota of Women Who Experienced Fetal Death in Utero
Source: Microorganisms. 2023 Jul 25;11(8):1877. doi: 10.3390/microorganisms11081877 (PMC10456767; doi:10.3390/microorganisms11081877)
Supplement: Supplementary file 1 [file microorganisms-11-01877-s001.zip › Supplementary Material Figures S1 & S2.pdf]

Supplementary Material Figures

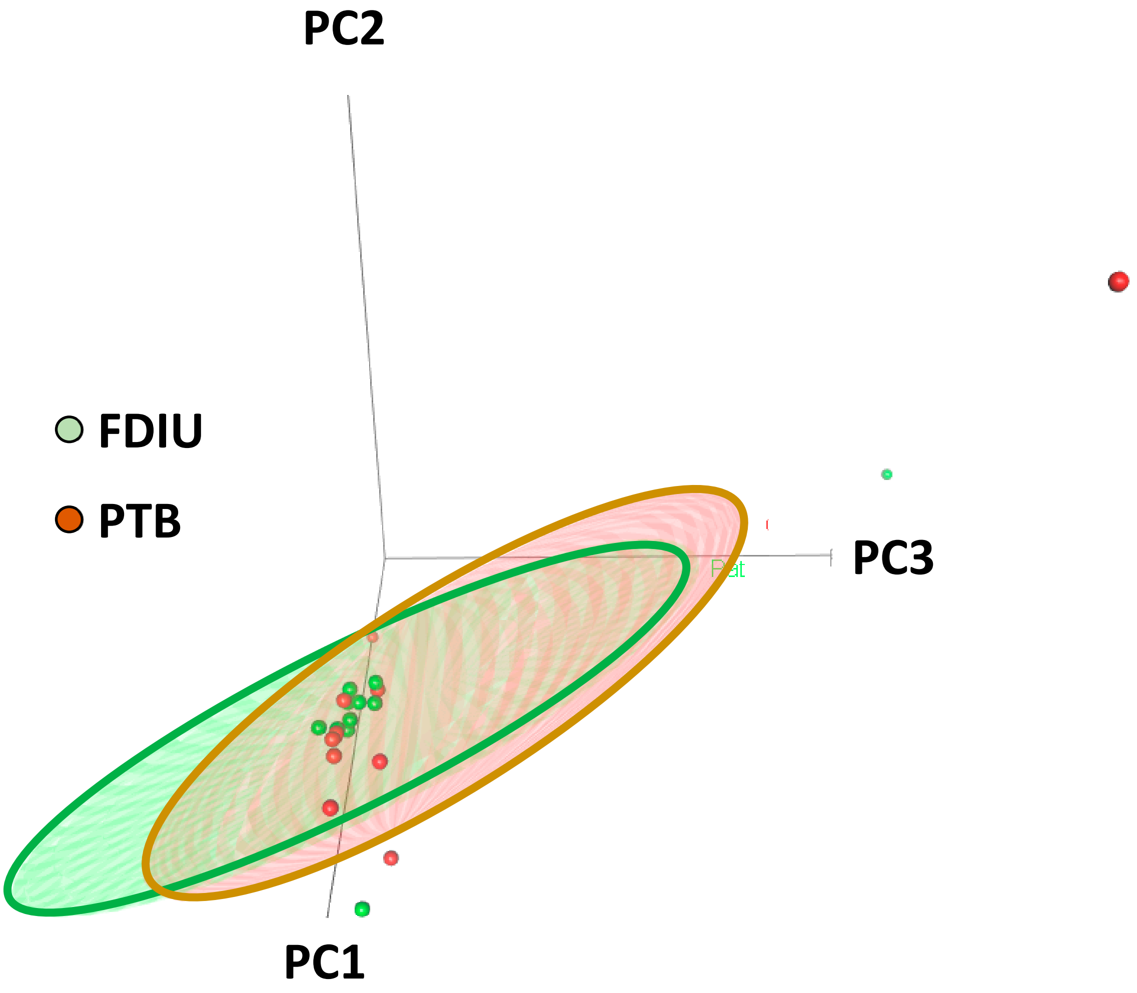

Figure S1. Principal Component Analysis of the taxa relative abundances comparing the composition of the vaginal microbiomes of women who delivered stillbirth (green dots) and PTB (brown dots). The ellipsoid contours enclose 50% of the data of each group.

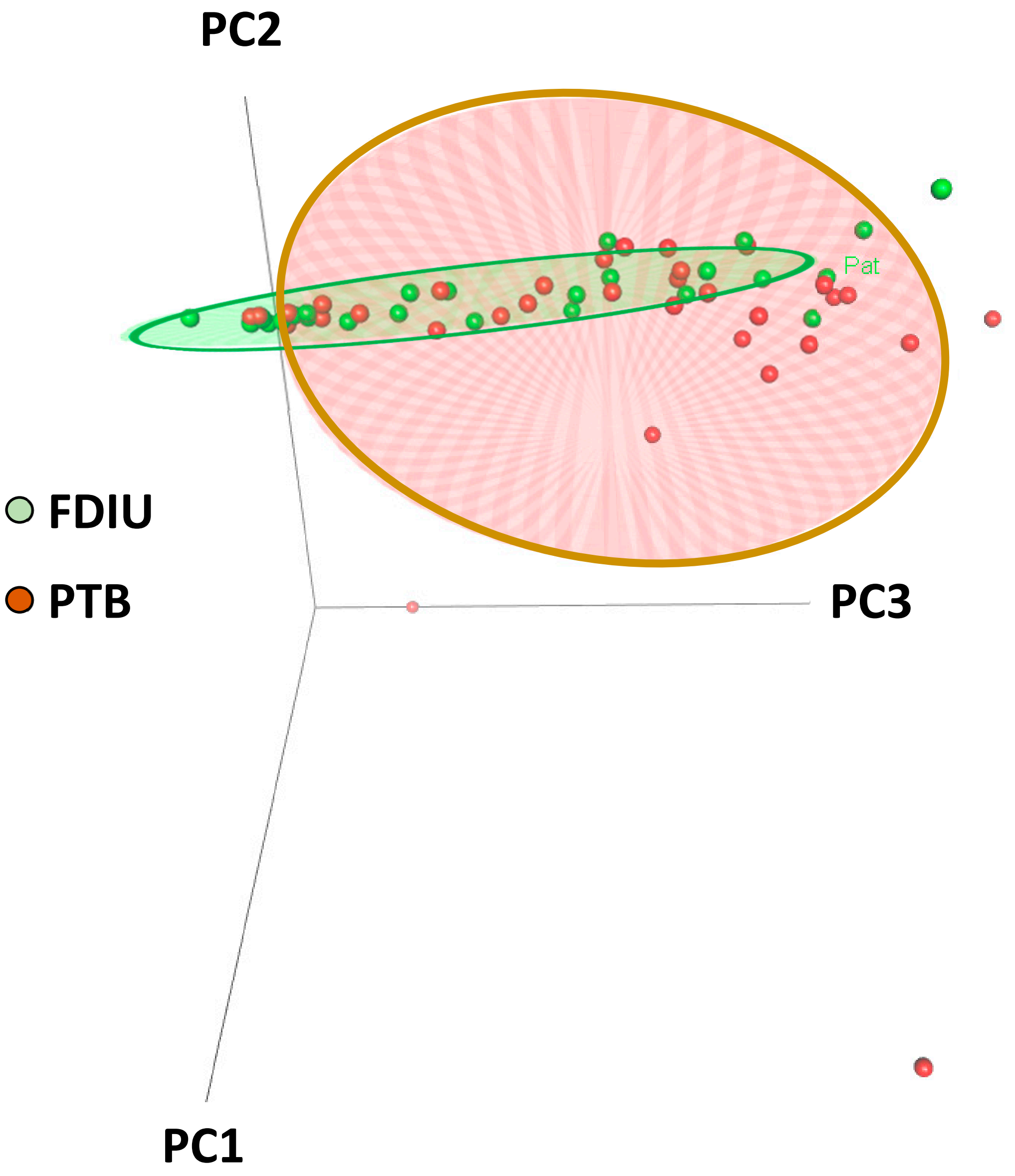

Figure S2. Principal Component Analysis of the taxa relative abundances comparing the composition of the amniotic microbiomes of women who delivered stillbirth (green dots) and PTB (brown dots). The ellipsoid contours enclose 50% of the data of each group.
